# Ultrasound-assessed diaphragmatic impairment is a predictor of outcomes in patients with acute exacerbation of chronic obstructive pulmonary disease undergoing noninvasive ventilation

**DOI:** 10.1186/s13054-018-2033-x

**Published:** 2018-04-27

**Authors:** Alessandro Marchioni, Ivana Castaniere, Roberto Tonelli, Riccardo Fantini, Matteo Fontana, Luca Tabbì, Andrea Viani, Francesco Giaroni, Valentina Ruggieri, Stefania Cerri, Enrico Clini

**Affiliations:** 1 Respiratory Diseases Unit and Centre for Rare Lung Diseases, Department of Medical and Surgical Sciences, University of Modena Reggio Emilia, University Hospital of Modena, Modena, Italy; 2 University Hospital of Modena, School of Medicine, University of Modena Reggio Emilia, Modena, Italy

**Keywords:** Diaphragmatic dysfunction, Noninvasive ventilation, Respiratory failure, Transdiaphragmatic pressure, Ultrasound

## Abstract

**Background:**

Ultrasound (US) evaluation of diaphragmatic dysfunction (DD) has proved to be a reliable technique in critical care. In this single-center prospective study, we investigated the impact of US-assessed DD on noninvasive ventilation (NIV) failure in patients with acute exacerbations of chronic obstructive pulmonary disease (AECOPD) and its correlation with the transdiaphragmatic pressure assessed using the invasive sniff maneuver (Pdi sniff).

**Methods:**

A population of 75 consecutive patients with AECOPD with hypercapnic acidosis admitted to our respiratory intensive care unit (RICU) were enrolled. Change in diaphragm thickness (ΔTdi) < 20% during tidal volume was the predefined cutoff for identifying DD+/− status. Correlations between ΔTdi < 20% NIV failure and other clinical outcomes were investigated. Correlation between ΔTdi and Pdi sniff values was analyzed in a subset of ten patients.

**Results:**

DD+ patients had a higher risk for NIV failure than DD− patients (risk ratio, 4.4; *p* <  0.001), and this finding was significantly associated with higher RICU, in-hospital, and 90-day mortality rates; longer mechanical ventilation duration; higher tracheostomy rate; and longer RICU stay. Huge increases in NIV failure (HR, 6.2; *p* < 0.0001) and 90-day mortality (HR, 4.7; *p* = 0.008) in DD+ patients were found by Kaplan-Meier analysis. ΔTdi highly correlated with Pdi sniff (Pearson’s *r* = 0.81; *p* = 0.004). ΔTdi < 20% showed better accuracy in predicting NIV failure than baseline pH value and early change in both arterial blood pH and partial pressure of carbon dioxide following NIV start (AUCs 0.84 to DTdi < 20%, 0.51 to pH value at baseline, 0.56 to early change in arterial blood pH following NIV start, and 0.54 to early change in partical pressure of carbon dioxide following NIV start, respectively; *p* < 0.0001).

**Conclusions:**

Early and noninvasive US assessment of DD during severe AECOPD is reliable and accurate in identifying patients at major risk for NIV failure and worse prognosis.

## Background

Patients admitted to the intensive care unit (ICU) or respiratory intensive care unit (RICU) because of severe episodes of acute exacerbations of chronic obstructive pulmonary disease (AECOPD) have considerably high in-hospital (24%) and 1-year (59%) mortality rates [1–3]. The use of noninvasive ventilation (NIV) in patients experiencing respiratory failure due to AECOPD is considered a first-line treatment but still has a failure rate between 5% and 40% [4]. In particular, risk of death is much higher in those patients receiving invasive mechanical ventilation (MV) once an NIV strategy has proven to be ineffective [5].

Researchers in previous studies have observed that patients with chronic obstructive pulmonary disease (COPD) might have a higher rate of diaphragmatic dysfunction (DD) than to age- and sex-matched healthy control individuals [6]. During AECOPD, biological factors related to systemic inflammation, prolonged use of steroids, and lung mechanical abnormalities due to hyperinflation may act as synergic mechanisms leading to DD [7]. Dynamic hyperinflation during AECOPD worsens the level of end-expiratory lung volume and residual volume, thus shifting tidal volume (V_t_) toward the right side of the pressure-volume curve [8]. As a result, higher intrathoracic pressures are needed to maintain an adequate V_t_. Furthermore, the early collapse of terminal airways with air entrapment causes an intrinsic positive end-expiratory pressure (PEEPi) that behaves as an adjunctive load that the respiratory muscles must overcome before generating inspiratory flow [7].

In a recently published pilot study, we reported that DD as assessed by a noninvasive ultrasound (US) technique is present in almost one-fourth of patients with AECOPD and admitted to the RICU for severe hypercapnic respiratory failure [9]. Although it is recognized that lung hyperinflation might play a critical role in DD, the impact of this derangement on AECOPD course and treatment remains incompletely elucidated. Therefore, in the present study, we wanted to investigate the clinical outcomes of patients with AECOPD requiring NIV and presenting with DD detectable by US. NIV failure was the prespecified primary outcome, and secondary outcomes were RICU, in-hospital, and 90-day mortality rates; duration of MV; incidence of tracheostomy; and RICU and in-hospital lengths of stay. Moreover, in these patients, we aimed at correlating the DD as assessed by US with the transdiaphragmatic pressure capacity (Pdi) as measured at maximal inspiration using the sniff maneuver (Pdi sniff).

## Methods

### Study population

This prospective observational cohort study was carried out in a single six-bed RICU at the University Hospital of Modena (Italy) over a 24-month period (January 2015 to January 2017). Approval from the local ethics committee of Modena was obtained (registered protocol number 839/C.E.). Written informed consent to participate was obtained from all patients enrolled or from their relatives, when appropriate.

Eligible patients older than 18 years of age were those consecutively admitted for acute acidotic hypercapnic respiratory failure following AECOPD and requiring NIV. Exclusion criteria were any of the following: presence of acute pulmonary edema, coexistence of interstitial lung disease, history of neuromuscular disease, chest wall deformities, previously assessed diaphragmatic palsy, shock or severe hemodynamic instability, intracranial hypertension, known pregnancy, and/or need for immediate endotracheal intubation. All patients were treated according to the best current clinical practice by the attending staff in the RICU, who were blinded to the purpose of the study.

### NIV treatment

NIV was started and set by an expert physician. Patients were not sedated and were connected via a face mask (Philips Respironics, Murrysville, PA, USA) to a high-performance ventilator (Engström Carestation; GE Healthcare Life Sciences, Helsinki, Finland) in pressure preset mode. External positive end-expiratory pressure was initially set to 5 cmH_2_O and subsequently fine-tuned according to clinical parameters and ventilator waveforms. Pressure support was set to 10 cmH_2_O and then progressively increased, according to V_t_, MV, and waveforms in order to obtain a V_t_ of 8–10 ml/kg and a respiratory rate of < 30 breath/min. The inspiratory fraction of inspired oxygen (FiO_2_) was increased to achieve a transcutaneous saturation of 88–94%. The setting was adjusted by the attending physician on the basis of blood gases and/or continuous oximetry.

NIV was delivered as long as possible on day 1, then for 16 h/day and 12 h/day on days 2 and 3, respectively. NIV was then discontinued on day 4 on the basis of clinical judgment or within the first 3 days in case of low compliance or clinical deterioration. Patients for whom NIV trials failed were switched to endotracheal intubation. The decision whether to perform endotracheal intubation or tracheostomy was made by the attending physician according to recommendations [10], but blinded to the result of the assessment of diaphragmatic function.

### General measures

On admission, clinical severity was recorded as the Kelly scale score, Acute Physiology and Chronic Health Evaluation II (APACHE II) score, and Simplified Acute Physiology Score II (SAPS II). Arterial blood gases (partial pressure of arterial oxygen [PaO_2_], partial pressure of arterial carbon dioxide [PaCO_2_]), pH, PaO_2_/FiO_2_ ratio, and respiratory rate were recorded at baseline and at least 2, 4, 6, 12, 24, and 48 hours following NIV. Blood lactate was measured on admission and then repeated if needed. The presence of pneumonia or sepsis [11], previous treatment with systemic steroids, forced expiratory volume in 1 second in the previous 6 months, stage of COPD, and relevant comorbidities were recorded.

### US assessment

US assessment of the diaphragm was performed on admission and before starting NIV by a respiratory physician with high expertise in lung/chest US evaluation. Motility of the diaphragm was assessed with a B-mode US device at the bedside (GE Vivid 7, GE Healthcare, Little Chalfont, UK) connected to a 7- to 12-MHz linear probe. Measurements were performed with the patient in supine position with an average inclination of 45 degrees. The position of the probe was set to obtain the best view of the zone of apposition of the diaphragm, located between the midaxillary and the posterior axillary lines. The diaphragm was identified as a three-layer structure consisting of one relatively nonechogenic muscle layer coated in two echogenic lines determined by peritoneal serosa and diaphragmatic pleura. Diaphragm thickness was measured bilaterally at end inspiration and end expiration. Images were stored in electronic or paper format by an examiner unaware of the study purpose. The change in diaphragm thickness (ΔTdi) during spontaneous breathing from functional residual capacity (FRC) to V_t_ was calculated as follows [12]:

$$ \Delta \mathrm{Tdi}=\left(\mathrm{end}\ \mathrm{inspiration}\ \mathrm{Tdi}-\mathrm{end}\ \mathrm{expiration}\ \mathrm{Tdi}/\mathrm{end}\ \mathrm{expiration}\ \mathrm{Tdi}\right)\times 100\ \left(\mathrm{thickening}\ \mathrm{fraction}-\mathrm{TF}\right) $$

Measurements were performed three times on both sides of the diaphragm. The best value as representative of the diaphragm function was then recorded for analysis.

The presence of DD (DD+) was defined according to the presence of ΔTdi bilaterally less than 20% (ΔTdi at most < 20%), as previously reported [13]. The accuracy of US in identifying DD+ and diaphragmatic paralysis was assessed through comparison with available measurements by sniff maneuver (*see below*).

### Transdiaphragmatic pressure assessment

In a limited number of highly collaborative patients, additional esophageal pressure (Pes) and gastric pressure (Pga) levels were recorded using a commercially available balloon catheter (NutriVent® nasogastric polyfunctional catheter; SIDAM, Mirandola, Italy) before starting NIV. Catheters were positioned through the nares after induction of topical anesthesia using standard techniques [14] and connected to a pressure transducer (OptiVent monitor; SIDAM). Transdiaphragmatic generating pressure capacity (Pdi) was obtained by subtracting Pes from Pga (Pga – Pes) during a sniff maneuver. The correct positioning of the catheters was verified by checking the negative deflection of the Pes signal and positive deflection of the Pga signaling during a maximum inspiration maneuver. Positioning was also confirmed by obtaining a chest x-ray showing the location of the reference points on the esophageal and gastric balloon. An occlusion test was performed to assess the validity of Pes measurements [15]. Pdi was evaluated during maximal inspiratory maneuvers [13] as obtained through a sniff maneuver (Pdi sniff) starting at FRC. Measurements were repeated three times or until three of them varied by less than 20%; the best value was considered representative of Pdi sniff.

### Statistical analysis

The Prism 7.0 statistical software package (GraphPad Software, Inc., La Jolla, CA, USA) was used for analysis. A power test was performed (α = 0.05; power, 80%) considering a 23% prevalence of DD among patients with COPD [12], and a sample size of 75 patients was required to confidently perform analysis on the prespecified primary outcome (NIV failure). Descriptive statistics for continuous variables were presented as mean ± SD or associated to interquartile range. The nonparametric Wilcoxon test (Mann-Whitney) and Student’s *t* test were used for comparison of continuous variables. Associations between dichotomous variables were performed using the χ^2^ test or Fisher’s exact test, where appropriate. The correlation among NIV failure and ΔTdi < 20% as compared with baseline pH < 7.25, pH, and PaCO_2_ changes within the first 2 hours of ventilation was assessed through ROC analysis. The impact of DD+ or DD− on NIV failure and mortality over time was assessed by Kaplan-Meier survival function estimates. The accuracy of US measures in identifying DD+ was calculated with contingency analysis, and the correlation between ΔTdi and Pdi sniff was assessed through Pearson’s correlation coefficient. A *p* value less than 0.05 was considered significant.

## Results

**Fig. 1:**
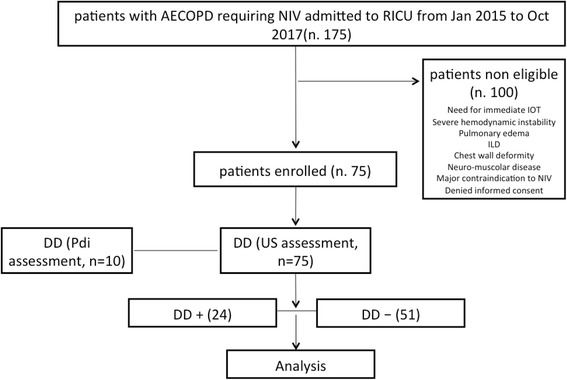
Study population diagram. AECOPD, *NIV* Noninvasive ventilation, RICU, *ILD* Interstitial lung disease, IOT, DD, Pdi, *US* Ultrasound

In the considered period, 75 of 175 patients with COPD admitted to our RICU for acute exacerbation requiring NIV treatment were enrolled and studied. According to the predefined criterion (best ΔTdi < 20%), 24 patients (32%) presented with DD at US (DD+), whereas 51 patients (68%) did not (DD−) (Fig. 1). The general features and clinical characteristics at baseline of the whole population and according the presence/absence of DD are presented in Table 1. All the patients enrolled were classified as GOLD (Global Initiative for Chronic Lung Disease) stage 4D when earlier in stable state [16]. The use of systemic steroids in the year preceding admission was the only variable that significantly differed between DD+ and DD− patients. The pressure-generating properties of the diaphragm (Pdi sniff), as recorded in a subset of ten patients, was highly reduced in DD+ patients as compared with DD− patients (19 mmHg [IQR 6–28] and 82 mmHg [IQR 77–87], respectively).

**Table 1:** Baseline characteristics of the study population as a whole and according to the presence/absence of diaphragmatic dysfunction

		Diaphragmatic function		
Feature	Overall	DD+	DD−	*p* Value
Patients	75 (100%)	24 (32%)	51 (68%)	
Age, years	78 (71–86)	77 (71–86)	78 (76–83)	n.s. (0.61)
Male sex	38 (51%)	15 (63%)	23 (45%)	n.s. (0.21)
Pneumonia	39 (52%)	14 (58%)	25 (50%)	n.s. (0.45)
Sepsis	23 (31%)	10 (42%)	13 (25%)	n.s. (0.1)
Diabetes	31 (41%)	10 (42%)	21 (41%)	n.s. (0.81)
Use of steroids	45 (46%)	17 (71%)	17 (33%)	0.005
FEV_1_	47% (30–65)	43% (27–61)	49% (32–67)	n.s. (0.65)
Kelly scale score	3.4 (2.4–4.1)	3.7 (2.9–4.3)	3.2 (2.5–3.7)	n.s. (0.34)
APACHE II score	22 (16–29)	25 (18–32)	20 (16–23)	n.s. (0.09)
SAPS II	43 (35–53)	47 (40–55)	41 (33–50)	n.s. (0.28)
PaO_2_/FiO_2_	166 (121–198)	165 (109–196)	168 (135–188)	n.s. (0.86)
pH	7.24 (7.2–7.3)	7.24 (7.21–7.29)	7.25 (7.19–7.36)	n.s. (0.32)
PaCO_2_, mmHg	91 (77–100)	91 (77–98)	90 (80–102)	n.s. (0.82)
Blood lactate, mg/dl	10 (5–12)	11 (4–12)	9 (5–10)	n.s. (0.72)
Respiratory rate, breaths/min	31 (29–35)	34 (30–36)	30 (28–35)	n.s. (0.07)

*Abbreviations*: *DD* Diaphragmatic dysfunction, *FEV*_*1*_ Forced expiratory volume in 1 second, *APACHE II* Acute Physiology and Chronic Health Evaluation II, *SAPS II* Simplified Acute Physiology Score II, *PaO*_*2*_*/FiO*_*2*_ Ratio of partial pressure of arterial oxygen to fraction of inspired oxygen, *PaCO*_*2*_ Partial pressure of arterial carbon dioxide

Data are presented as number and percent for dichotomous values or mean and IQR for continuous values

Table 2 shows the clinical outcomes in the study population. DD+ patients presented a higher risk for NIV failure than DD− patients (risk ratio 4.4; *p* < 0.001). Six DD+ patients who failed NIV then died, and twelve underwent endotracheal intubation. After intubation, two died while on MV, five underwent tracheostomy (two of whom died), and five were successfully weaned. Among DD− patients for whom NIV failed, four DD− patients for whom NIV failed then died, and four underwent endotracheal intubation (two died while on MV, and two underwent tracheostomy with one of them dying).

**Table 2:** Clinical outcomes (primary and secondary) of the study population

	Diaphragmatic function				
Outcome	Overall	DD+	DD−	Relative risk	*p* Value
NIV failure	26 (35%)	18 (75%)	8 (16%)	4.4 (2.3–8.7)	< 0.0001
RICU mortality	16 (21%)	10 (42%)	6 (12%)	3.1 (1.3–7.7)	0.007
In-hospital mortality	19 (25%)	11 (46%)	8 (16%)	2.7 (1.3–5.7)	0.02
90-day mortality	29 (39%)	14 (58%)	15 (29%)	1.8 (1.1–3.1)	0.04
Tracheostomy	7 (9%)	5 (21%)	2 (3.9%)	5 (1.2–21)	0.04
MV duration, days	10 (3–11)	16 (5.5–18.8)	8 (2–9)	2 (1.4–3.3)	0.03
ICU stay, days	14 (6–17)	17 (8–21)	12 (7–16)	2.8 (1.5–4.2)	0.012
Hospital stay, days	21 (10–23)	21 (10–23)	22 (10–24)	1.1 (0.5–1.3)	n.s. (0.9)

*Abbreviations*: *DD* Diaphragmatic dysfunction, *NIV* Noninvasive ventilation, RICU Respiratory intensive care unit, *MV* Mechanical ventilation

Data are presented as number and percent for dichotomous values or mean and IQR for continuous values

Among the secondary outcomes, DD+ correlated with higher short- and long-term mortality, longer stay in the RICU, prolonged MV, and higher tracheostomy rate (Table 2).

**Fig. 2:**
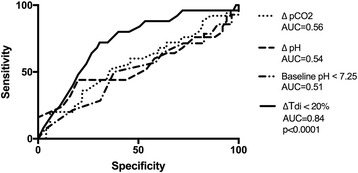
ROC analysis comparing predictors for noninvasive ventilation (NIV) failure at baseline and within 2 hours after NIV was started. *ΔTdi* Change in diaphragm thickness

**Fig. 3:**
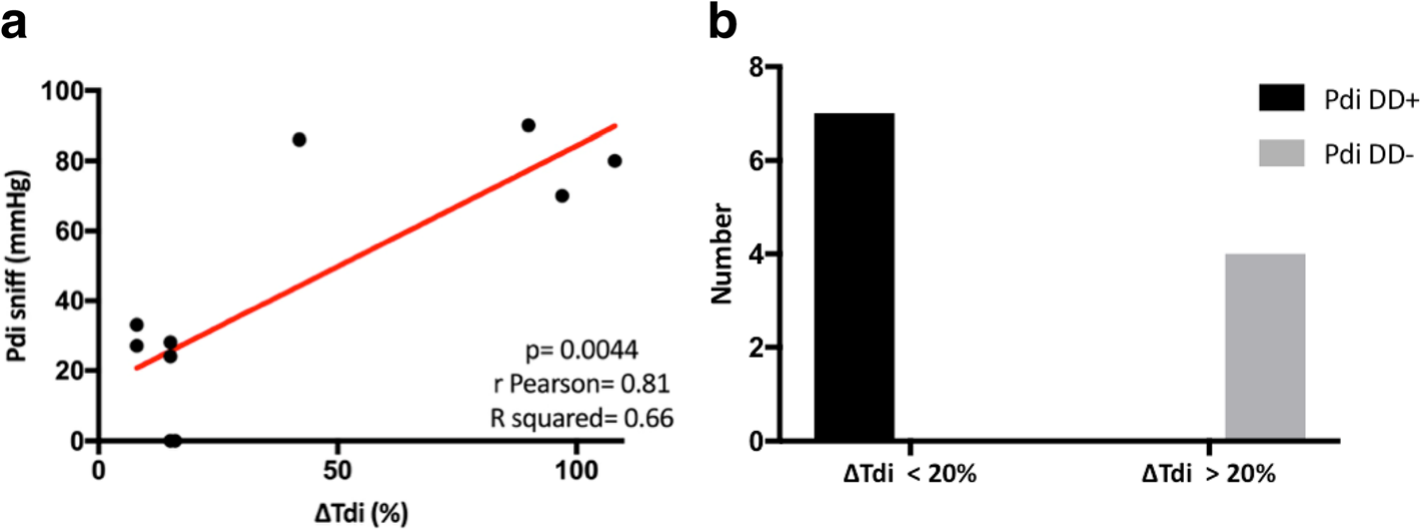
**a** Correlation between change in diaphragm thickness (ΔTdi) and transdiaphragmatic pressure capacity measured at maximal inspiration using the sniff maneuver (Pdi sniff). **b** Accuracy of ΔTdi and Pdi sniff in identifying patients with diaphragmatic dysfunction (DD)

In ROC analysis, ΔTdi < 20% showed higher accuracy in predicting NIV failure than baseline pH < 7.25, and both changes in arterial blood pH and PaCO_2_ did within 2 h after NIV was started (Fig. 2) (AUCs 0.84, 0.51, 0.56, and 0.54, respectively; *p* < 0.0001). In addition, ΔTdi showed a very good correlation with Pdi sniff (Pearson’s *r* = 0.81; *p* = 0.004) (Fig. 3a); moreover, ΔTdi < 20% demonstrated the same accuracy as Pdi sniff in identifying DD+ (sensitivity 100%; 95% CI 0.6–1; specificity 100% 95% CI 0.51–1; *p* = 0.0048) (Fig. 3b).

**Fig. 4:**
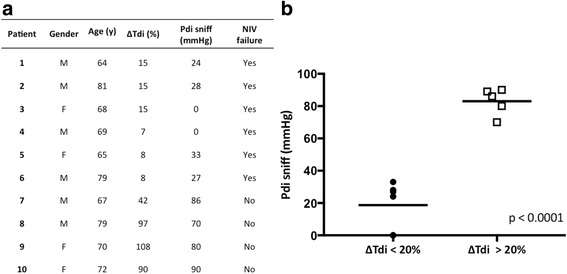
**a** Change in diaphragm thickness (ΔTdi) values at ultrasound testing and transdiaphragmatic pressure capacity measured at maximal inspiration using the sniff maneuver (Pdi sniff) values in the subgroup of ten patients tested with esophageal and gastric balloons. **b** Distribution of patients with ΔTdi < 20% or > 20% according to Pdi sniff. *NIV* Noninvasive ventilation

**Fig. 5:**
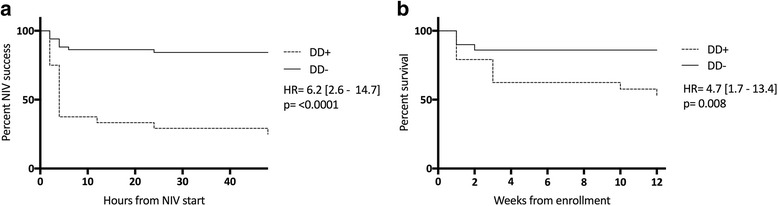
Probability of fail noninvasive ventilation (NIV) failure (**a**) and death (**b**) within the first 48 hours after admission according to the presence (+)/absence (−) of diaphragmatic dysfunction (DD) as assessed by ultrasound

Figure 4 reports ΔTdi and Pdi sniff values in the subgroup of ten patients tested with esophageal and gastric balloons (Fig. 4a) and distribution of patients with ΔTdi < 20% or > 20% according to Pdi sniff (Fig. 4b). Among patients with ΔTdi < 20%, two patients had a Pdi sniff of 0, meaning complete diaphragm paralysis, whereas four presented Pdi sniff values between 24 and 33 cmH_2_O. Regarding diaphragm paralysis, ΔTdi demonstrated high sensitivity but low specificity in detecting this condition (sensitivity 100%, 95% CI 0.18–1; specificity 50%, 95% CI 0.22–0.78). Kaplan-Meier curves showed a significant increase in NIV failure and 90-day mortality (Fig. 5a and b, respectively) among DD+ versus DD− patients.

## Discussion

In this single-center prospective observational study, we show that patients DD+ on their admission day in the RICU had a sixfold increased risk of NIV failure within the first 48 hours and an almost fivefold higher risk of dying during the follow-up. In addition, DD+ was associated with increased RICU and hospital lengths of stay, prolonged use of MV, and higher tracheostomy rate. Finally, the presence/absence of DD as described by the ΔTdi level (namely the *thickening fraction*) at US testing was strongly correlated with the diaphragmatic pressure-generating capacity as assessed by Pdi sniff. Thus, the early and noninvasive assessment of DD during severe AECOPD requiring NIV seems accurate and helpful in avoiding the risk of delayed intubation in patients with this critical condition.

Our study demonstrates a significant prevalence of DD in patients with severe AECOPD (32%). In a previous exploratory study on the same subject [12], we found that DD similarly assessed by US was reported in 22% of total patients; however, that sample had fewer patients and less severity than the present study (APACHE II score 20 vs 22, *p* = 0.041; and SAPS II score 41 vs 43, *p* = 0.036).

Demoule et al. reported that the prevalence of DD, as assessed by twitch tracheal pressure in response to bilateral anterior magnetic phrenic nerve stimulation, was 64% in a heterogeneous group of patients admitted to a general medical ICU [5]. However, the two populations seem difficult to compare because the population in our present study is limited to a specific subset of critical patients. Moreover, we used a more restricted definition of DD, the ΔTdi in the zone of apposition less than 20%, thus representing a likely condition of very severe impairment, even confirmed by the transdiaphragmatic Pdi assessment as obtained in ten subjects.

The physiopathological mechanism(s) underlying the onset of DD during AECOPD is multifactorial. However, the synergic effect of mechanical disadvantage, functional (exhaustion) and biological (inflammation) impairment, and pharmacological damage (steroid-induced) could only be postulated.

In patients with COPD with frequent exacerbations the maximum pressure produced by the diaphragm’s contraction is significantly than in individuals without COPD, in the same regard as the volitional (Pdi at total lung capacity and/or Pdi sniff) or nonvolitional (phrenic nerve stimulation) nature of the test applied [17–19]. This difference has been explained by the diaphragmatic shortening and the mechanical derangement following onset of progressive lung hyperinflation [17]. Indeed, patients with severe COPD produce higher transdiaphragmatic pressures at equivalent lung volumes than healthy control individuals [20]. The reason for this imbalance has been found in increased airway resistance, reduced dynamic lung elastance, and raised PEEPi, which are related to the progressive air trapping [17, 20]. Thus, in a patient with AECOPD, the expiratory flow limitation and the increased respiratory rate with reduced expiratory time favor the onset of hyperinflation with subsequent respiratory muscle shortening and limited excursion. The reduction in muscle strength seems even more inadequate to cover the excessive mechanical load imposed by the increased respiratory rate. In these conditions, the diaphragm soon exhausts its functional reserve, and ultimately mechanical impairment occurs.

In the present study, exposure to steroids in the previous year was the only feature differentiating DD+ from DD− patients. To date, no studies have been conducted in humans to investigate the specific effect of steroid course on the respiratory muscles during AECOPD. Notwithstanding this, studies performed on the skeletal muscles clearly show an increase in proteolysis and a reduction in the expression of insulin-like growth factor (IGF)-1 following the use of steroids [21]. In animal models, there are studies showing damage to the diaphragmatic structure after administration of systemic steroids [22, 23], with proteolysis and downregulation of IGF-1 being the plausible mechanisms responsible for steroid-induced myopathy [24]. Therefore, it is possible that previous or current (ab)use of systemic steroids could have contributed to the development of respiratory muscle weakness, thus leading to DD once AECOPD occurred.

Even despite the lack of a preadmission assessment of diaphragmatic function, some aspects seem to indicate that dysfunction might be related to the current acute condition rather than to a chronic exposure in our study population. First, the use of a very low cutoff limit of ΔTdi (< 20%) to identify DD+ let us individuate patients with a condition close to muscle paralysis, whose presence would already have been evident outside the acute phase [25]. Second, studies conducted with patients with stable severe COPD did not identify a significant difference in diaphragmatic function by US evaluation at V_t_ compared with healthy individuals [26]. Third, in a study by Orozco-Levi et al., transdiaphragmatic pressure values were similar in patients with stable COPD and normal control subjects, thus indicating a substantial functional preservation, although regional stresses and strains (muscle geometry, increased workload during exertion, mechanical stress, and metabolic factors) might induce or accentuate diaphragmatic injury [27].

It is likely that these potential mechanisms of damage are amplified during AECOPD, when the diaphragm is called on to sustain stresses much greater than during clinical stability. Our study demonstrates a positive and significant correlation between ΔTdi at US assessment and Pdi measurement at maximal inspiration. To our knowledge, this is the first time that US evaluation of the diaphragmatic function has been compared with standard techniques in subjects with AECOPD.

Kim et al. showed that US is reliable in identifying DD during weaning from MV and in estimating the work of breathing during NIV [28]. Gottesman et al. indicated that ΔTdi < 20% is the cutoff limit for identifying diaphragm paralysis [25]. In our study, only two patients had Pdi sniff equal to 0 among those with ΔTdi < 20% (see Fig. 4). The sensitivity and specificity of ΔTdi (< 20%) to detect DD+ dropped to 50% in cases of diaphragm palsy.

Overall, the present study demonstrates that US is able to identify dysfunction but not palsy of the diaphragm, for which invasive maneuvers are needed. Given that these patients usually present with acute hypercapnic respiratory failure, the use of volitional or invasive techniques such as transdiaphragmatic pressure might become extremely challenging, in contrast to US assessment.

The main finding of our study is that DD+ patients, once confirmed by US, present a greater than fourfold risk of NIV failure during severe AECOPD (*see* Fig. 5). Interestingly, the two slopes start separating significantly 4–6 hours after starting NIV, then both groups gradually display a plateauing distribution over time.

Several studies have consistently demonstrated that the severity of hypercapnia and acidosis are associated with early NIV failure during AECOPD [13, 29–31]; however, no studies have assessed the impact of DD on NIV outcome. Indeed, our previous exploratory study demonstrated only that DD+ is related to NIV failure in a small population underpowered to test this hypothesis [9].

In the present study, we were able to demonstrate that DD+ as assessed by US may predict NIV failure with higher accuracy than both baseline pH < 7.25 and early change of pH and PaCO_2_ (*see* Fig. 2). Notwithstanding this, the reasons for the exceeding NIV failure rate are not completely clear, whereas severe expiratory flow limitation and excessive hyperinflation might have played a critical role in uncoupling patients’ efforts and MV. Because the use of NIV is increasing in different hospital settings, the present data may be of help to select patients with AECOPD who might be successfully ventilated in a general ward (patients without DD), rather than those who would benefit by being treated with MV in the ICU (patients with DD). Therefore, this finding increases the clinical importance of early identification of patients at greater risk for NIV failure even in the ward [32].

In our patients with AECOPD and DD+ short-term (in the RICU) and 90-day mortality are up to fivefold greater than in DD− patients. This finding is in line with data reported by Demoule et al., who described a higher mortality rate in patients admitted to the ICU with low tracheal pressure in response to bilateral anterior magnetic phrenic nerve stimulation [5]. In that hospital setting, a potential explanation for increasing death rate following DD has been identified in the early impairment of nerve conduction and mitochondrial alterations due to the onset of systemic inflammation [33].

Our present data suggest only that the duration of ventilation and the prolonged stay in the critical area following NIV failure may have resulted in a worse prognosis. Interestingly, we did not find any significant difference in hospital stay between patients with impaired or preserved diaphragmatic function. However, it could be hypothesized that other independent factors might have influenced the length of admission (e.g., home setting, chronic comorbidities, lack of domestic facilities).

Our study provides new information on the pathophysiology of AECOPD needing NIV treatment; however, it has some limitations that need to be addressed. First, it was conducted in a single center, which means that the results need to be verified in a multicenter study. Because we performed a prespecified sample size analysis for the primary outcome, the findings at least warrant further investigations. Second, DD by US assessment was performed during spontaneous breathing in a critical care setting without any evaluation of lung volumes. Because different volumes highly correlate with the ability of the diaphragm to contract, measurement of volumes might have helped us to better understand the overall mechanism leading to progressive hyperinflation and then to DD. Third, we did not reassess diaphragmatic function over time, so data regarding potential spontaneous recovery are lacking. Last, we did not investigate the inflammatory status (i.e., the circulation levels of cytokines) in our patients, though this has been supposed to be a pathway of damage in the diaphragm (*see paragraph above*).

## Conclusions

In this single-center trial, we observed that patients with severe AECOPD admitted to the RICU for NIV treatment have a significantly higher risk of failure and mortality when DD, as noninvasively assessed by US, occurs. US measurement of DD definitely correlates with transdiaphragmatic pressure-generating capacity in these subjects. Therefore, early evaluation of the diaphragm by US in this setting may help clinicians to identify patients with AECOPD at major risk for a negative prognosis.

## Abbreviations

AECOPD: Acute exacerbation of chronic obstructive pulmonary disease
APACHE II: Acute Physiology and Chronic Health Evaluation II
COPD: Chronic obstructive pulmonary disease
DD: Diaphragmatic dysfunction
FiO_2_: Fraction of inspired oxygen
FRC: Functional residual capacity
IGF-1: Insulin-like growth factor 1
MV: Mechanical ventilation
NIV: Noninvasive ventilation
PaCO_2_: Partial pressure of arterial carbon dioxide
PaO_2_: Partial pressure of arterial oxygen
Pdi sniff: Transdiaphragmatic pressure capacity measured at maximal inspiration using the sniff maneuver
Pdi: Transdiaphragmatic pressure
PEEPi: Intrinsic positive end-expiratory pressure
Pes: Esophageal pressure
Pga: Gastric pressure
RICU: Respiratory intensive care unit
SAPS II: Simplified Acute Physiology Score II
US: Ultrasound
V_t_: Tidal volume
ΔTdi: Change in diaphragm thickness
